# Acceleration of chronic wound healing by bio-inorganic polyphosphate: *In vitro* studies and first clinical applications

**DOI:** 10.7150/thno.67148

**Published:** 2022-01-01

**Authors:** Hadrian Schepler, Meik Neufurth, Shunfeng Wang, Zhengding She, Heinz C. Schröder, Xiaohong Wang, Werner E.G. Müller

**Affiliations:** 1 Department of Dermatology, University Clinic Mainz, Langenbeckstr. 1, D-55131 Mainz, Germany.; 2 ERC Advanced Investigator Grant Research Group at the Institute for Physiological Chemistry, University Medical Center of the Johannes Gutenberg University, Duesbergweg 6, D-55128 Mainz, Germany.; 3 Shenzhen Lando Biomaterials Co., Ltd., Building B3, Unit 2B-C, China Merchants Guangming Science Park, Guangming District, Shenzhen 518107, China.; 4 NanotecMARIN GmbH, Mühlstr. 19, D-55218 Ingelheim am Rhein, Germany.

**Keywords:** Inorganic polyphosphate, Nanoparticles, Chronic wounds, Compressed collagen, Re-epithelialization

## Abstract

The healing of chronic wounds is impaired by a lack of metabolic energy. In previous studies, we showed that physiological inorganic polyphosphate (polyP) is a generator of metabolic energy by forming ATP as a result of the enzymatic cleavage of the high-energy phosphoanhydride bonds of this polymer. Therefore, in the present study, we investigated whether the administration of polyP can substitute for the energy deficiency in chronic wound healing.

**Methods:** PolyP was incorporated into collagen mats and applied *in vitro* and to patients* in vivo*.

**Results: *(i) In vitro* studies:** Keratinocytes grown *in vitro* onto the polyP/collagen mats formed long microvilli to guide them to a favorable environment. HUVEC cells responded to polyP/collagen mats with an increased adhesion and migration propensity as well as penetration into the mats. ***(ii) In vivo -* human clinical studies:** In a “bench to bedside” process these promising *in vitro* results were translated from the laboratory into the clinic. In the proof-of-concept application, the engineered polyP/collagen mats were applied to chronic wounds in patients. Those mats impressively accelerated the re-epithelialization rate, with a reduction of the wound area to 65% after 3 weeks and to 36.6% and 22.5% after 6 and 9 weeks, respectively. Complete healing was achieved and no further treatment was necessary. Biopsy samples from the regenerating wound area showed predominantly myofibroblasts. The wound healing process was supported by the use of a polyP containing moisturizing solution.

**Conclusion:** The results strongly recommend polyP as a beneficial component in mats for a substantial healing of chronic wounds.

## Introduction

Exact numbers for wound care, including chronic wounds (venous leg ulcers, diabetic foot ulcers, and pressure ulcers) remain unknown 1. In the United States, around 15% of all Medicare beneficiaries (8.2 million) suffer from at least one type of wound or wound infection. After surgical wound infections (4.0%), diabetic wound infections have the second highest prevalence with 3.4%. With a focus on annual cost expenditures, wound care estimates range between $30 and $100 billion 2. The costs for chronic wounds account for ~1-3% of the total healthcare expenditures in industrialized countries 3.

**Figure 1:**
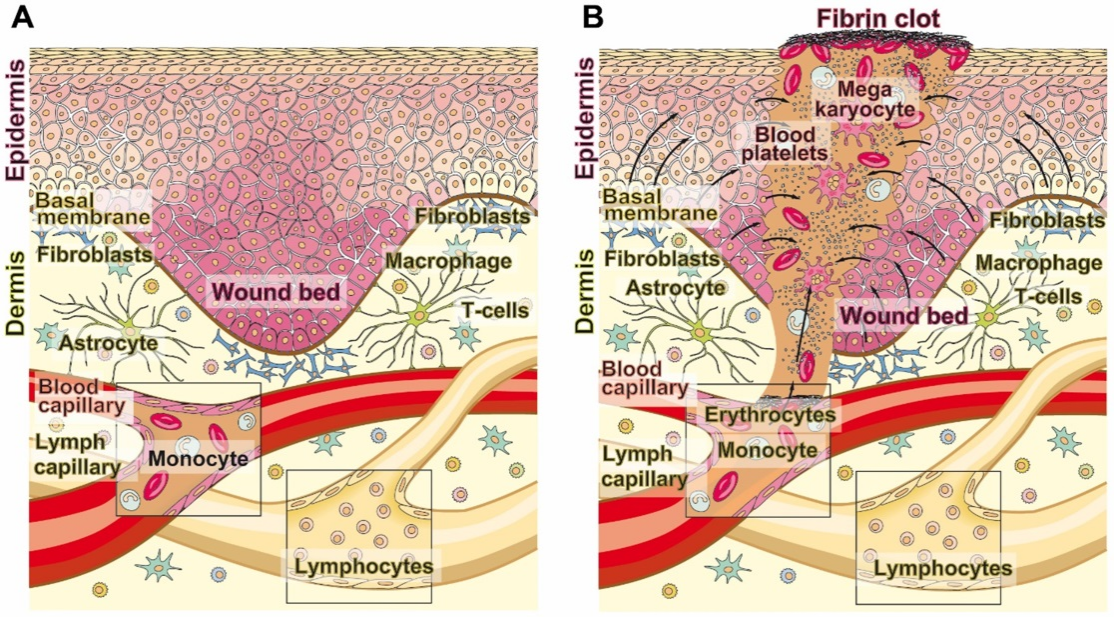
Wound healing. (**A**) The adult human skin organ consists of an epidermis and a dermis layer. While the epidermis is built of keratinocytes that are layered in four distinct strata, from basal cell layers to prickle and granular to horny ones. The dermis is composed of collagenous, reticular, and elastic fibers and primarily comprises fibroblasts but also immune cells (macrophages, astrocytes, and T-cells). The dermis is interspersed with blood and lymph capillaries. (**B**) When blood capillaries are wounded and damaged, platelets originating from megakaryocytes infiltrate into the wound area (also including the epidermis) and initiate blood clotting through a series of activation processes. The fibrin clot is formed, which seals the damaged tissue. During re-epithelialization and angiogenesis the extracellular matrix is reconstituted.

The skin with the epidermis as the outermost layer acts as a physical barrier against hazardous environmental physical irradiation as well as inorganic or organic noxae and bacteria. Wounds disrupt the epidermis and often the dermis, including the blood vessel system (Figure 1A and B). As a result metabolically active blood cells migrate into the damaged tissue and cause a hypoxic environment 4. If, in parallel with this process, systemic conditions such as advanced age or diabetes exist, which impair the vascular flow, the hypoxic situation is even exacerbated. In chronic wounds that are particularly hypoxic, a low transcutaneous tissue oxygen tension of 5-20 mm Hg (controls, 30-50 mm Hg) can be measured. Frequently, chronic wounds fail to proceed through the normal phases of wound healing 5. The lack of wound repair, of re-epithelialization, leads both to a loss of the barrier function of the organ and to dehydration, infection or even death.

Wound healing, both repair and regeneration, incomplete or complete, is divided into ordered and overlapping phases; hemostasis, inflammation, proliferation, and maturation 6. This programmed process is under the control of external as well as internal stimuli, growth factors and cytokines. A breakdown of the progression of these phases leads to impairment of normal wound repair, frequently to the development of nonhealing, chronic wounds 7. The delivery of the repair factors (chemokines, cytokines, matrix molecules, or nutrients) to the damaged region and of inflammatory cells requires an intact blood vessel system. These processes are compromised in diabetes 8, ischemia and with increasing age 9. The common denominator of these changes is a decreased metabolic energy supply to the defects, which affects the rate of wound healing, parallel to a delayed or absent wound regeneration, based on a lack of blood nutrients and low growth factor supply 10,11.

The oxygen demand in wounds is higher than in healthy tissue, which is also reflected in an increased ATP consumption during the different phases of skin regeneration. The metabolic energy is needed for the processes of collagen/protein synthesis and both for cell proliferation and for angiogenesis 12. The energy supply of the cells in the wound area covers only about 10% of the energy required for wound regeneration.

Wound care is essential in view of the increase in chronic wounds and the associated morbidity in the developed countries 7. In the first step of wound care, necrotic tissue is removed by surgically debridement or enzymatic autolysis. A slightly delayed application of inorganics (such as silver) or antibiotics is often indicated. Subsequently, the wound is protected from bacterial infections, often by a moist occlusive dressing, which also provides a humid environment with a low oxygen tension that favours increased re-epithelialization 13. During the inflammation, proliferation, and maturation phases wound dressings are used to cover the defect and support wound healing. Covering with conventional wound dressings such as dry gauze has the inherent risk of micro-injuries during removal. Therefore, electrospun low adherent and semipermeable films have been developed that restrict liquid and microbial penetration and simultaneously allow air and water vapour permeability 14. An advancement came with the fabrication of hydrocolloid and hydrogel coverages that equally heal dry wounds and remove necrotic tissue 15. Finally, collagen-based products have been introduced as dressing to cover persistent wounds and chronic ulcers.

As indicated, the supply of metabolic energy is crucial for successful wound healing. In the context of the data mentioned, it is established that malnutrition or deficiency/depletion in carbohydrates, protein, fatty acids or vitamins impairs the wound healing process 16. A major challenge for the further development of active wound dressings is therefore the identification of an energy source and its subsequent formulation in the wound dressing. The main metabolite that feeds the energy-consuming regeneration processes in the body is ATP. The concentration of this nucleotide in the extracellular space increases at the beginning of wound repair 17. ATP acts as a signalling molecule for the cells that respond with epidermal growth factor (EGF)-like growth factor shedding and EGF receptor transactivation. In continuation, the energy-carrying ATP has been applied either directly intracellularly in wounds 18 or in a precursor form, also extracellularly, as polyphosphate (polyP) 19,20. PolyP is, like bio-silica, an inorganic biomaterial that is endowed with distinguished biological properties 21,22,23.

Focusing on polyP, the therapeutic results shown *in vivo* are promising. This polymer was applied for wounds as nanoparticles (NP). When used in the NP form, as calcium polyphosphate nanoparticles (Ca-polyP-NP) 24,25, a significant acceleration in the progress of wound healing was seen in mice. In studies with diabetic mice, the re-epithelialization rate even doubled compared to untreated animals 20. Indicative were the results of histological examinations, which showed that the formation of granulation tissue increases dramatically in wounds treated with polyP-NP 20. Within the newly formed connective tissue, microvessels developed. In order to proof the assumption that polyP positively affects vascularization, *in vitro* studies with the tube forming assay have been performed. The data showed that during incubation of HUVEC (human umbilical vein endothelial cells) with polyP a burst of ATP is generated in the extracellular space, which is paralleled with an intensified tubule formation 26. This rearrangement of endothelial cells was completely blocked by addition of the ATP consuming apyrase. In a subsequent detailed analysis it was shown that inhibitors of alkaline phosphatase (ALP; levamisole) and adenylate kinase (ADK; Ap_5_A [P^1^,P^5^- di(adenosine-5')pentaphosphate]) completely block ATP generation and tubule formation 27. The concerted action of these enzymes has been implicated in the process of ATP generation from polyP 21,28. The ALP channels the released energy from the polyP anhydride bond to AMP to form ADP. In the second step, two moles of ADP are converted into one mole each of ATP and AMP.

In the present study a wound dressing is described, which is supplemented with polyP, and subsequently applied to HUVEC, a model cell system for angiogenesis 29, using the *in vitro* wound scratch assay. In addition, human keratinocytes were used to quantify cell attachment and invasion of the cells into the mats. Finally, the collagen-based mats were used as a proof-of-concept *in vivo* in patients with chronic wounds according to the established recommendations 30. The results demonstrate that the polyP-supplemented material is not only biocompatible for the cells and increases their activity with regard to migration and invasion *in vitro*, but also strongly accelerates the healing of chronic wounds in patients. The clinical application was performed in a translational approach. So far, a total of five patients suffering from chronic ulcerations of the lower extremities have been successfully treated, initially, in the frame of an off-label use. The data collected show that polyP is a promising component for a successful treatment and even healing of chronic wounds.

## Results

### Preparation and *in vitro* application of the collagen mats

#### Preparation of collagen-based mats

**Figure 2:**
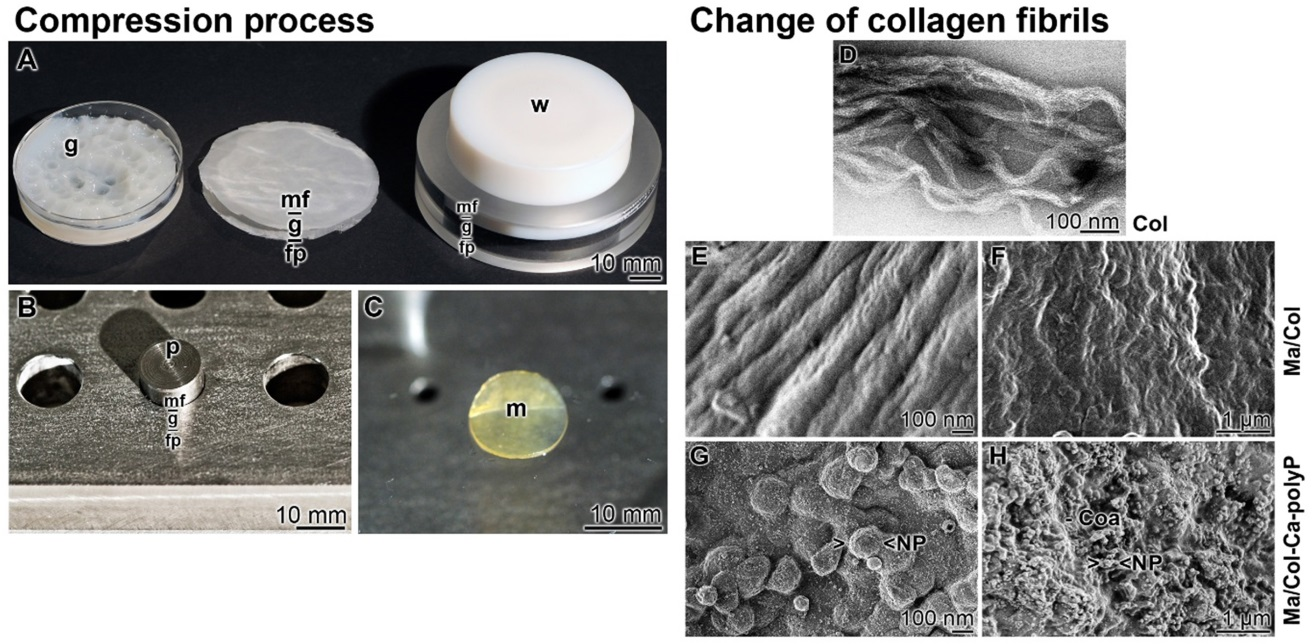
** Stabilized collagen-based mats. (A to C)** The compression process. (**A-Left**) Collagen type I was suspended in acetic acid at pH 3.6. The gel-like material (g) was poured into a Petri dish and neutralized. (**A-Middle**) The gel pad (g) was layered onto a filter paper stack (fp) and overlayed with a nylon mesh filter (mf). (**A-Right**) The gel-like material was pressed with a weight (w) to an ~1 mm thick collagen mat, the sequential arrangement of the nylon mesh filter (mf) - gel pad (g) - filter paper stack (fp) is only hinted. (**B**) Stainless steel mat press. Into the cavities the gel-like collagen material was filled in and placed onto a filter paper. The upper side was covered with the mesh filter and a weight of 80 g was put on the punch (p). (**C**) The pressed 1 mm thick collagen mat (m). **(D to H)** Change of morphology of the collagen fibrils during the processing phases; SEM. (**D**) Poorly-formed collagen subfibrils at pH 3.6. (**E**) Transformation of the subfibrils to mature D-banded collagen fibers at pH 6 to 9 in “Ma/Col”. (**F**) Pressed collagen fibers in the 1 mm thick mats. (**G and H**) Surface of “Ma/Col-Ca-polyP-NP” with nanoparticles (NP) that are concentrated on the ridges of the D-banded collagen fibers. (**H**) Surface appearance of “Ma/Col-Ca-polyP-NP” mats with their NP, which are embedded into the coacervate phase of polyP (Coa).

The bovine tendon collagen type I was prepared in a solvent of pH 3.6. This suspension was pH neutralized in a Petri dish (diameter of 50 mm) during which a gel-like layer is formed. In order to increase the stability, the 3-5 mm thick film was pressed with a weight of 20 g for 20 min. By this, an about 1 mm thick mat is formed (Figure 2A). While the collagen-based mats with a diameter of 50 mm are used for human wounds, small size mats were prepared in small scale in a stainless steel mat press (Figure 2B). The prepared 8 mm sized collagen mats had likewise a thickness of 1 mm (Figure 2C).

#### Change of collagen assembly

The organization of collagen fibrils is polymorph and dependent on the pH milieu 31,32.

At pH 3.6 (in acetic acid) the soluble collagen type l molecules interact loosely and form poorly-formed collagen subfibrils (Figure 2D). After transfer of the subfibrils to pH 6 (citrate-phosphate buffer) and then to pH 9 (Tris buffer), mature D-banded collagen fibers are formed (Figure 2E). Squeezing the collagen fibers resulted in more dense mats of higher stability (Figure 2F). The surfaces of these “Ma/Col” mats are slightly ribbed (Figure 2F).

To enrich the mats with polyP, the collagen solution was supplemented with 8 mg of “Ca-polyP-NP” per 1 mL collagen suspension and processed; “Ma/Col-Ca-polyP-NP”. Finally the mechanically pressed mats were washed in PBS and then in ethanol (Figure 2G and H). At higher magnification the NP are arranged at the ridge of the D-banded collagen fibers 19 (Figure 2G). At lower magnification it becomes manifest that the NP are partially embedded in a coacervate matrix (Figure 2H).

### Propensity of keratinocytes/HUVEC to attach and migrate onto the “Ma/Col”/“Ma/Col-Ca-polyP-NP”

#### Attachment of keratinocytes onto collagen-based mats

**Figure 3:**
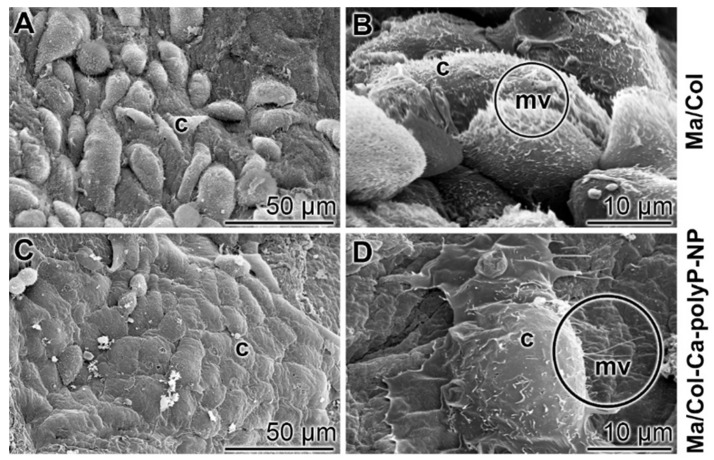
** Keratinocytes that readily attach to collagen-based matrices. (A and B)** Lower abundance of keratinocytes attached to the polyP-free matrix, “Ma/Col”, in contrast to cells (c) attached to (**C and D**) “Ma/Col-Ca-polyP-NP”. The matrices were overlayed with cells (4 • 10^4^ cells/mL) for 24 h. After incubation, images were taken by ESEM. Noticeable are the microvilli (mv; circled) developed by keratinocytes on “Ma/Col-Ca-polyP-NP”.

To study the attachment of keratinocytes onto the polyP-lacking “Ma/Col” and the polyP-enriched “Ma/Col-Ca-polyP-NP” hydrogel matrix, a keratinocyte cell suspension (4×10^4^ cells/mL) was overlayed onto the two formulations and incubated for 24 h in medium/serum. Then the samples were inspected by ESEM [environmental scanning electron microscopy] (Figure 3). In the cell system onto the “Ma/Col” matrix only comparably few cells attached to the surface (Figure 3A and B), while in the assays with “Ma/Col-Ca-polyP-NP” (Figure 3C and D) the surface is densely covered with cells. Impressive is the change in the morphology of the cells. The keratinocytes onto the “Ma/Col-Ca-polyP-NP” change to a 'cobblestone' pattern, which is characteristic for keratinocytes in the lowermost epidermal cell layer, the stratum basale 33; they are the interfollicular epidermis stem cells from where the clonogenic keratinocytes maturate 34 (Figure 3C and D). In addition and frequently, these cells are covered with numerous microvilli (Figure 3D). The length of the actin-rich microvilli undergoes shortening under adverse substrate conditions 35. The ESEM images show that the keratinocytes cultured onto “Ma/Col” are very short (Figure 3B), while they develop to mature microvilli on the polyP matrix (Figure 3D). The microvilli implement important functions as sensory organelles for sound, light, and odor perception 36.

#### Cell viability and migration of HUVEC cells onto the collagen-based matrices

##### Cell growth and proliferation

**Figure 4:**
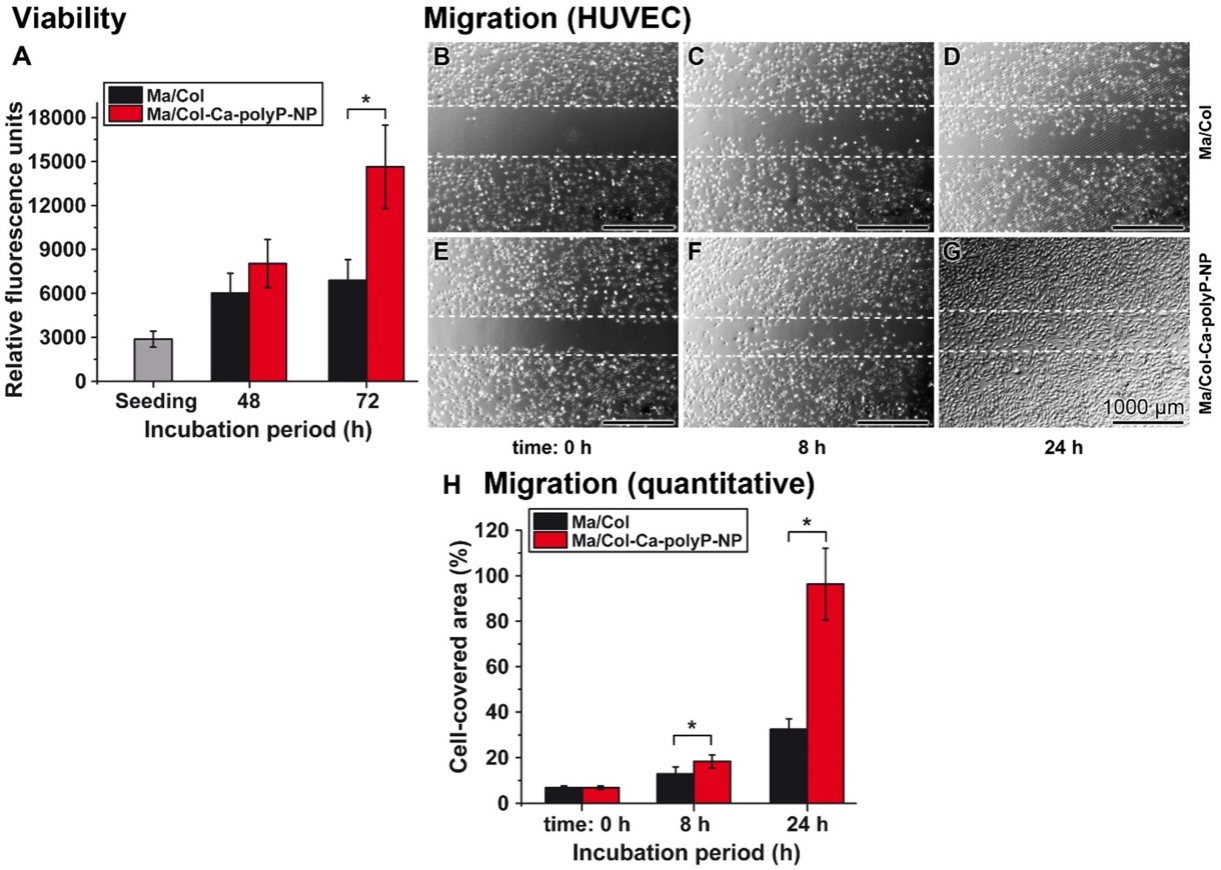
** Viability (A) and migration activity (B to H) of HUVEC onto the collagen-based substratum.** (**A**) The cells were seeded onto either “Ma/Col” or “Ma/Col-Ca-polyP-NP”. At day 0 (seeding) a color reaction caused by resorufin is measured with 2870±542 relative fluorescence units. After 48 h the cell viability between the two systems is not significantly different. However, after 72 h a strong difference between the two matrices is measured by 2.1-fold (*p <0.01). (**B to G**) Migration activity of HUVEC cells, as determined in the cell migration assay (scratch assay); n=5 (representative images are shown). It is impressive that the migration activity of the cells on the “Ma/Col-Ca-polyP-NP” substratum is exceeding by far that seen for the one on “Ma/Col”. The dashed lines mark the borders of the edges of the cells immediately after the scratch. (**H**) A computer-based evaluation shows that the difference between the migration activity of HUVEC onto “Ma/Col-Ca-polyP-NP” mats is already significantly higher after an incubation period of 8 h, compared to the cell migration propensity on “Ma/Col” mats. This difference is very pronounced after a 24 h incubation (n = 5;* *p* <0.01).

HUVEC were cultivated onto a 1 mm collagen-matrix layer with the two formulations. The mats remained non-supplemented as “Ma/Col” or were enriched with polyP as “Ma/Col-Ca-polyP-NP”. Applying the colorimetric cell viability PrestoBlue system a slight increase of the cell growth onto “Ma/Col” by 2.1- to 2.4-fold was measured during the 48 h or 72 h incubation period, in comparison to the density after cell seeding. In contrast, if the cells were plated onto “Ma/Col-Ca-polyP-NP” the growth/viability increased significantly by 5.1-fold during the 72 h incubation, compared to the assay during seeding (Figure 4A).

##### Cell migration

HUVEC cell layers grown to confluency were damaged by scratching. Then the assays were continued to be incubated for 8 h and 24 h, respectively. At time 0 h no cells were seen in the scratched zones (Figure 4B and E). If the incubation was continued for 8 h and especially for 24 h, the HUVEC migrated into the scratched area (Figure 4C and D, as well as F and G). Even more, the cells in the assays with “Ma/Col-Ca-polyP-NP” reached almost a continuum in the repaired scratch regions (Figure 4G). A quantitative evaluation revealed that the migration activity in the assays with “Ma/Col-Ca-polyP-NP” is already significantly higher after an incubation period of 8 h, compared to the cell migration propensity on the surface of “Ma/Col” (Figure 4H). The difference is very distinct, with 3.1-fold, after an incubation period of 24 h.

#### Attachment and morphology of keratinocytes onto “Ma/Col” and “Ma/Col-Ca-polyP-NP”

**Figure 5:**
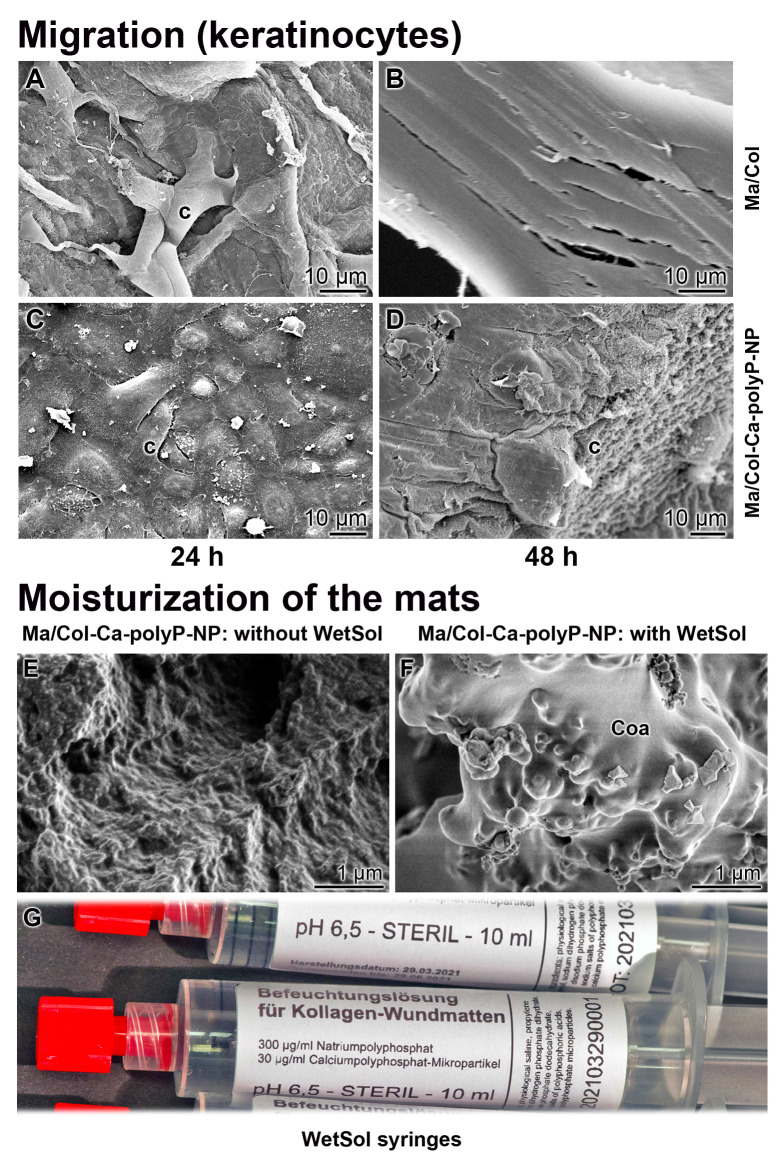
** Collagen-based mats and wetting solution. (A to D)** Migration propensity of keratinocytes on collagen-based mats. The cells were seeded either on (**A and B**) “Ma/Col” or on (**C and D**) “Ma/Col-Ca-polyP-NP” and incubated in medium/serum for 24 h or 48 h; ESEM. Cells, seeded on “Ma/Col” remain on the surface of the matrix (**A**) and do not migrate into the mat (**B**). In contrast, after incubation on “Ma/Col-Ca-polyP-NP” the cells (c) invade (**C**) from a dense layer on the surface of the mats into their lower layers (**D**) after a 48 h incubation period. Five parallel samples were analyzed. **(E to G)** Moisturizing of the wound with the sterile wetting solution “WetSol”. Collagen mat before (**E**) and (**F**) after moistening with “WetSol”. The treated, moisturized sample was kept in a humid chamber for 6 h and then processed for microscopic analysis (SEM). During this period a coacervate (Coa) layer is formed on the surface of the mat. (**G**) 10 mL of sterile “WetSol” were filled into 10 mL syringes fitted with a Luer-Lock-adapter.

Keratinocytes have the property of invading hydrogel matrices under suitable conditions 37. In the series summarized here it is shown that the cells seeded onto “Ma/Col” remain on the surface of the mats and show either not at all or only a slight propensity to invade “Ma/Col” during the 48 h incubation period (Figure 5A and B). In contrast, if the cells are plated onto “Ma/Col-Ca-polyP-NP” (Figure 5C and D), already after 48 h the cells are present within the “Ma/Col-Ca-polyP-NP” mats and appear there as cell clusters deep in the collagen matrix (Figure 5D).

### Preparation of the wetting solution

A curative wound care requires a humid environment for the regenerating dermis and epidermis 7. Therefore, a wetting solution “WetSol” was formulated, which contained in a pH buffered (pH 6.5) and physiological solution polyP, both in the soluble (Na-polyP) and the particulate form (Ca-polyP-NP). The application of this recipe onto “Ma/Col-Ca-polyP-NP” allowed the formation of a coacervate layer (Figure 5F), which is absent on the non-treated mat (Figure 5E). The suspension was supplied in the sterile form in 10 mL syringes fitted with a Luer-Lock-adapter (Figure 5G).

### *In vivo* curative effect of collagen-based mats

**Figure 6:**
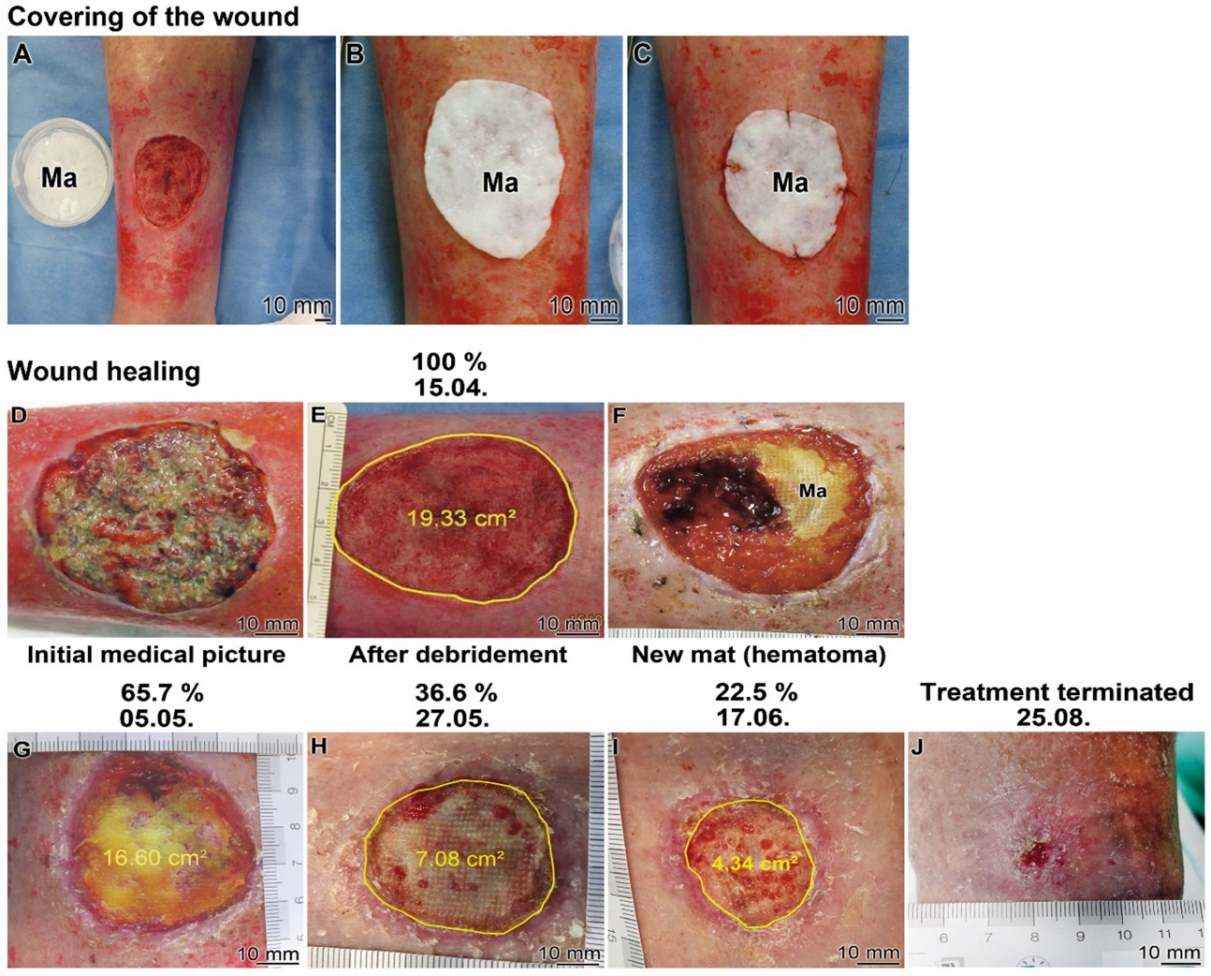
** Healing kinetics of a chronic wound (Patient 1) after application of the collagen-based mats, containing polyP; “Ma/Col-Ca-polyP-NP”. (A to C)** Covering of the chronic wound with a mat (Ma). (**A**) A sample of Ca-polyP-NP in a Petri dish is placed next to the wound. **(B and C)** Fixation of the mat on the wound**. (D to J)** Kinetics of wound healing. (**D**) Wound prior to debridement and (**E**) after this treatment. Above the images the time of taking the images is given (start of the treatment: 15.04.; the size of the wound which is circled within the image is given in percent above the corresponding image). (**F**) Occasionally, during the initial phase of healing, the collagen mat became overgrown by the epithelial layer next to hematoma regions. Progression of the healing after three weeks (**G**), six weeks (**H**) and nine weeks (**I**). After the 19 weeks medication the treatment could be terminated (**J**).

**Figure 7:**
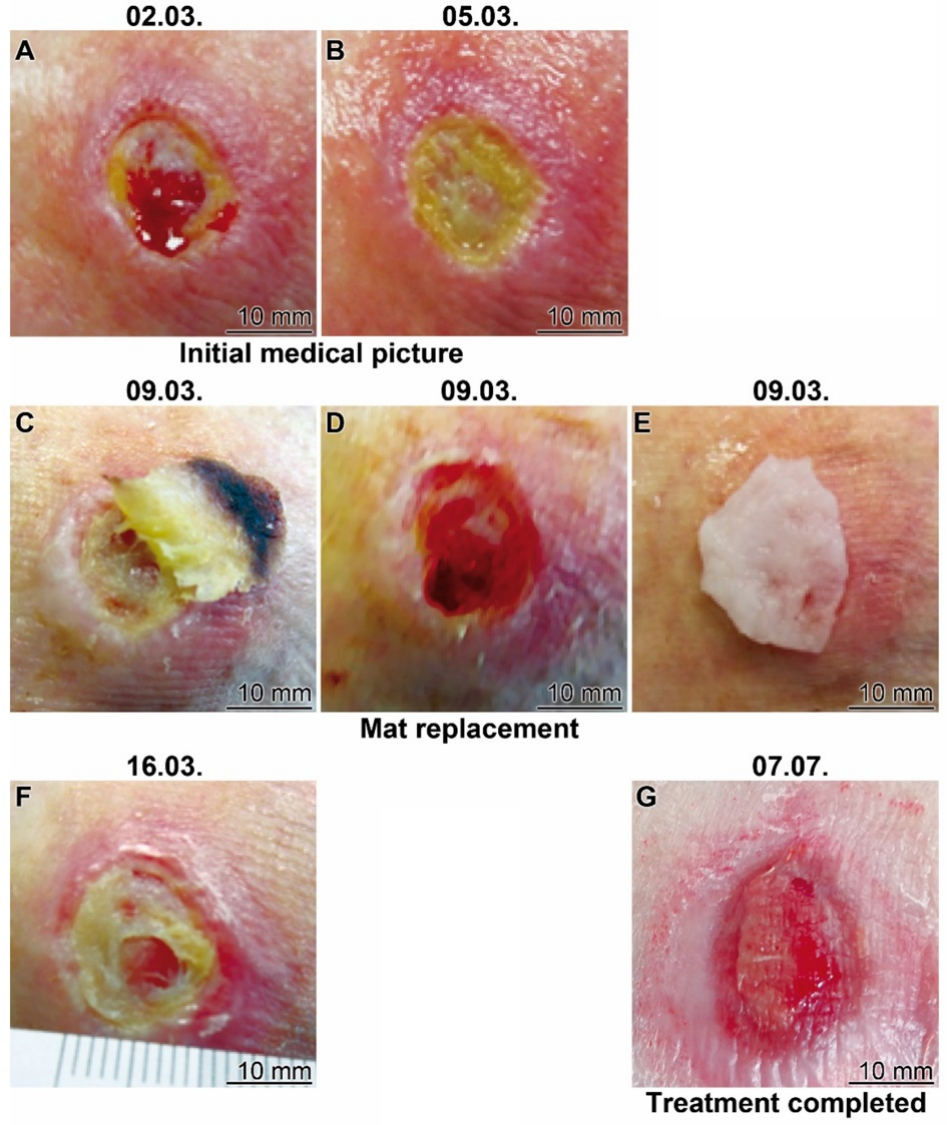
** Healing progress of a therapy resistant traumatic ulcer on the lateral malleolus (Patient 2). (A and B)** Initial clinical picture. The treatment started at 05.03. **(C to E)** After a four days' treatment the bulged mat was replaced (**C and E**) uncovering (**D**) a marginal epithelial rim around the wound. **(F)** The treatment was continued with continuous application of the wetting solution “WetSol”. **(G)** Ending of the treatment with the polyP-based mat and transition to a conventional covering with a conventional adhesive bandage.

In five off-label applications the collagen-based mats supplemented with polyP, “Ma/Col-Ca-polyP-NP”, were applied to patients with chronic wounds after frustrating standard therapies. Among them two representative cases are exemplified here (Figure 6 and Figure 7).

#### Patient 1

**Figure 8:**
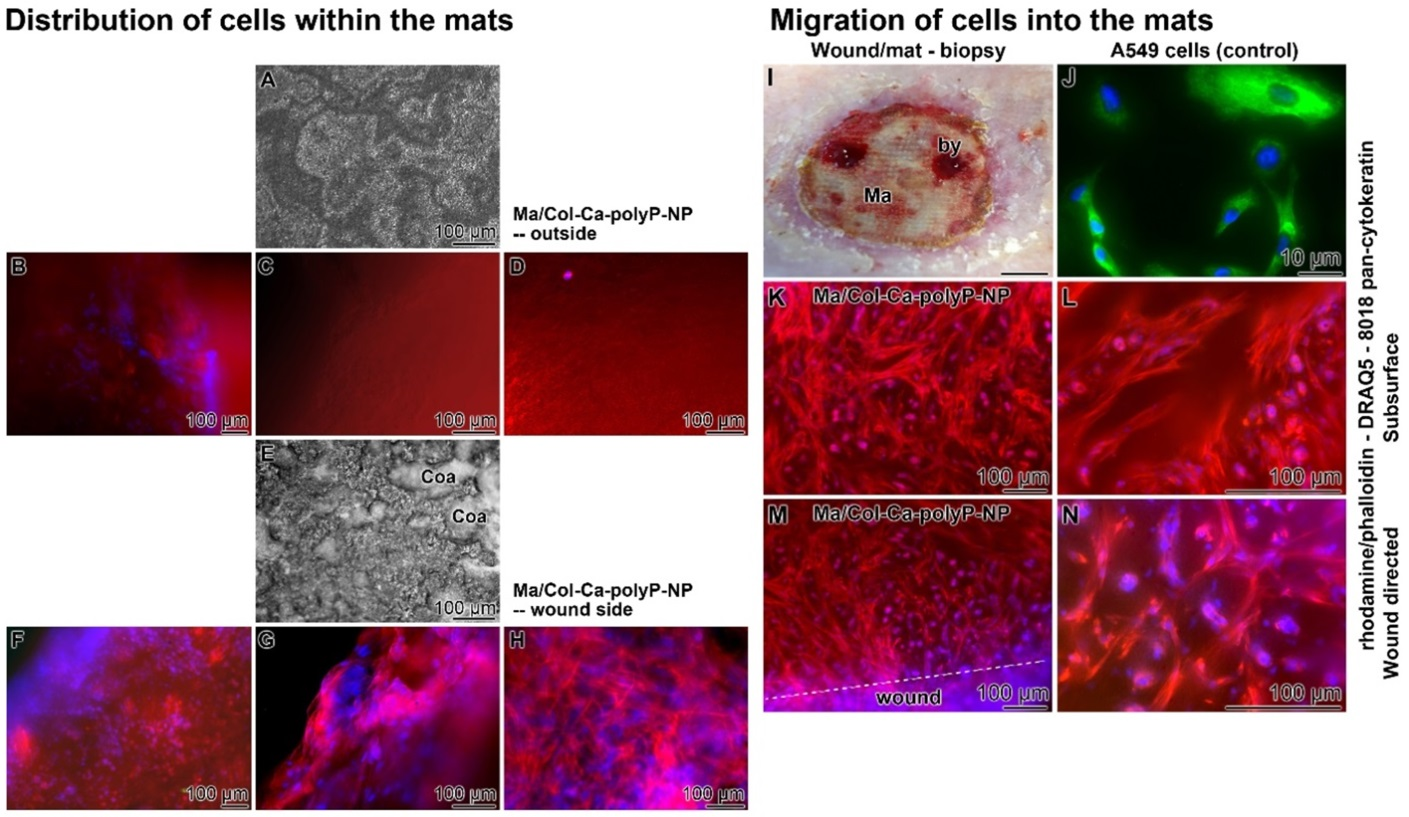
** (A to H) Distribution of cells within collagen-based mats placed onto chronic wound (Patient 1).** Rapid attachment of cells (after two days) onto “Ma/Col-Ca-polyP-NP” mats. (**A**) Outside oriented surface of the mat, lacking any patches of coacervate; (**E**) frequently present dense coacervate clusters (Coa) on the wound directed mat surface; light microscopy. (**B to D**) Absence of cells within the outer region of the mat; staining with rhodamine/phalloidin (red; for F-actin) - DRAQ5 (blue; nuclei); fluorescence microscopy. (**F to H**) Densely packed cells at the wound side area. **(I to N)** Migration of cells into the collagen mat. (**I**) Location of the biopsy (by) taken at the collagen mat (Ma) towards the granulation tissue of the regenerating wound (three weeks after starting the treatment); the biopsy was taken 7 days (after the last mat change) after covering the wound with “Ma/Col-Ca-polyP-NP”. (**J**) Human A549 epithelial cells show a bright staining in green for keratin. For staining of the cells from the patient (**K and L**) slices were taken from the biopsy at the subsurface region (250 µm oriented inwards), and also (**M and N**) from the immediate surface of the mat facing the wound. The sections were stained with rhodamine/phalloidin, together with DRAQ5 and the (sc-8018) pan-cytokeratin antibody. The cells highlight under fluorescent light only in red and blue, but are lacking any green fluorescence (no keratin).

**Figure 9:**
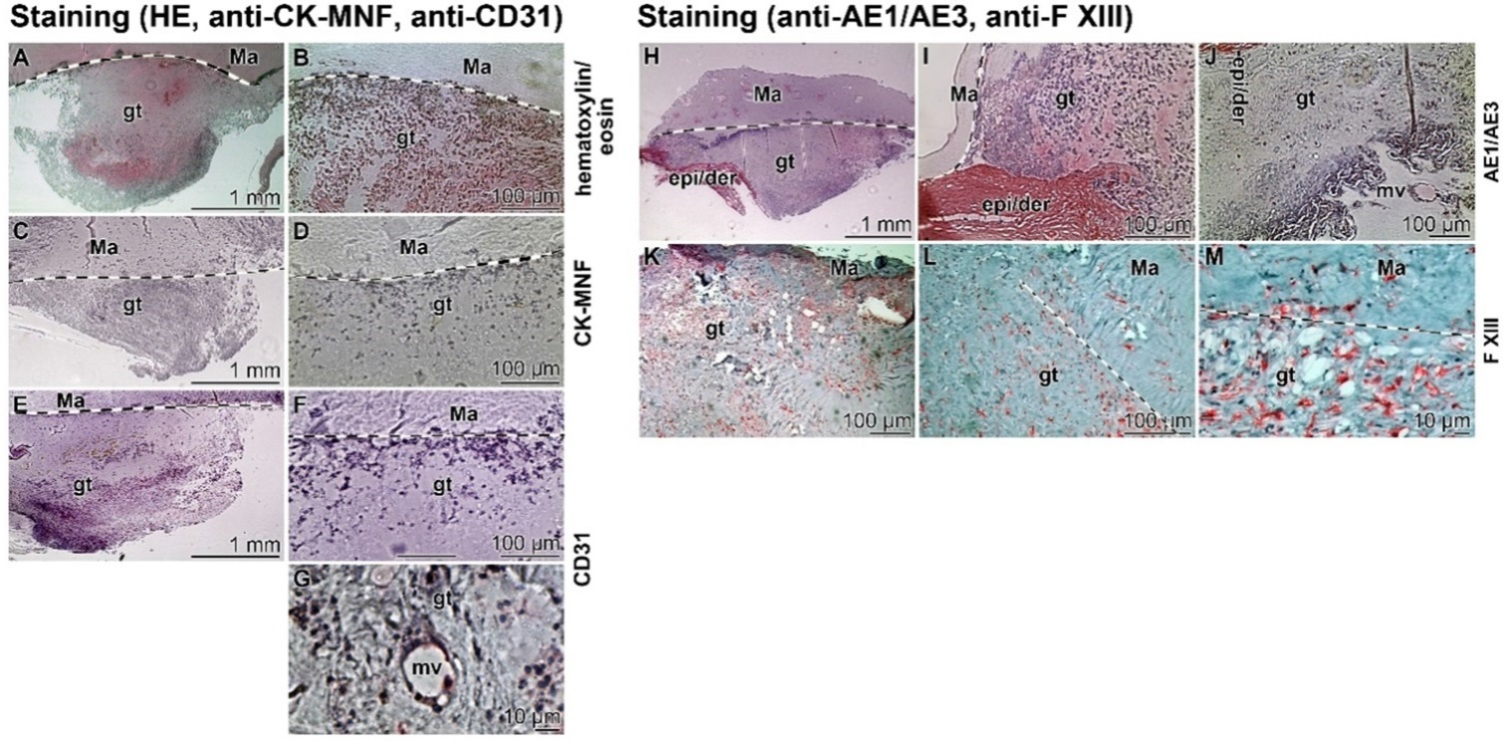
** Immunostaining of slices through a biopsy (Patient 1).** The boundaries between the collagen-based mat (Ma) and the regenerating granulation tissue (gt) are marked with spotted lines; light microscopy. **(A to G)** The tissue slices were stained with (**A and B**) hematoxylin/eosin, (**C and D**) cytokeratin pan antibody MNF116, and (**E-G**) anti-CD31 (PECAM-1) antibodies to highlight endothelial cells. In (**G**) the cells framing a microvessel (mv) and reacted with anti-CD31 are depicted. **(H-M)** Images from slices incubated with (**H-J**) an anti-cytokeratin AE1/AE3 antibody in comparison to those (**K-M**) reacted with a Factor XIII antibody.

The patient (79-years old male) developed a chronic wound after surgical resection of a squamous cell carcinoma. The wound healing treatment started in the hospital in April 2021 using the “Ma/Col-Ca-polyP-NP”. The wound was manifested as an ulcerative lesion. The ~20 cm^2^ large lesion (Figure 6A and D) was processed by surgery and ultrasound to remove the necrotic tissue and to allow surgical preparation of the wound bed prior to initiation of the wound care therapy with “Ma/Col-Ca-polyP-NP”. After cleaning of the wound (Figure 6E) the collagen-based wound dressing was applied to the defect on the ventral tibia of the left lower leg (Figure 6A). The mat, covering the wound (Figure 6B), was fixed (Figure 6C). The mat specimens covered the ~5 cm (in diameter) large chronic wound (Figure 6C and D). Subsequently, the regeneration progress of the wound was followed by sizing the healing defect (Figure 6E to J). The study was flanked by histological investigations (Figures 8 and 9).

At least every other day, the mat was moisturized with 1 mL of wetting solution, “WetSol”, in order to keep the wound moist and to support adherence of the dressing as well as to accelerate healing 38. The healing effect of “WetSol” is attributable to the polyP component in the suspension, which causes a coacervate layer onto the wound (Figure 5F). After two weeks the collagen mat was replaced with a new one to treat residual hematoma formed under the dressing (Figure 6F).

The healing kinetics is surprisingly fast. During the first three weeks the re-epithelization of the wound started centripetally from the wound rim and reduced the non-epithelialized wound area to 85.8% (to 16.6 cm^2^; Figure 6G). After a further treatment for three weeks the wound healed over to 36.6% (7.08 cm^2^; Figure 6H). Finally, after three more weeks almost a continuous epithelization was seen, which accounted for 22.5% (from the 100% wound area at the beginning of the study) of not completely healed up epidermis (Figure 6I). Only a few small, 1-3 mm large non-epithelialized regions remained. Treatment was terminated after a total of 19 weeks medication (Figure 6J). The recommendation for the patient was to cream the scar in order to smoothen this region. Only wound dressing changes were recommended after detailed instructions. Parallel with the (close to complete) reduction of the size of the wound, the patient reported a significant decrease in pain and he could keep his physical hobbies, like riding his bicycle.

#### Patient 2

The 82 old woman suffered from a therapy resistant traumatic ulcer on the lateral malleolus for one year. The therapy with the collagen-based and polyP-enriched mats, “Ma/Col-Ca-polyP-NP”, started in March 2021 (Figure 7). In the initial stage the wound is characterized by sloughy necrotic tissue and a crater-like depression; the patient complained about intense pain also intensified by the severe inflammation of the surrounding tissue (Figure 7A). The treatment with the polyP mats started (Figure 7B). After four days of application the mat (partially) slipped out (Figure 7C) and was subsequently replaced by a new polyP-enriched mat (Figure 7E). Already after this short period of time the peripheral redness strongly decreased and a marginal epithelial rim around the wound developed (Figure 7D). This change was paralleled with a reduction of pain. The healing of the wound continued (Figure 7F) and the size of the wound area decreased, disclosing centrally a stabilization of the granulation tissue. A regular application of the wetting solution to the wound area, “WetSol”, resulted in a complete filling of the former ulcer region with well perfused tissue and a thin epithelial layer, reflecting signs of a successful wound closure (Figure 7G). The treatment could be terminated and only a conventional band-aid was indicated.

### Distribution of cells in the collagen-based mats

The aim of the study on the distribution of cells (performed with patient 1) within the mats covering the wound was two-fold; first, to document the spreading of the cells on the two planes of the “Ma/Col-Ca-polyP-NP” mat, and second, to demonstrate their invasion propensity into the mat. Therefore, for studying the cell attachment the mat was removed from the wound after two weeks also due to a haematoma and replaced by another collagen mat. In the second series, the mat was left on the wound and then analyzed for cell infiltration after clinical integration by a permitted biopsy.

During the short-term coverage it was found that the outward-facing surface showed no signs of formation of a polyP-derived coacervate (Figure 8A), while the plane of the mat facing the wound was covered with coacervate spots (Figure 8E). After staining the specimens with rhodamine/phalloidin (distribution of the F-actin cellular cytoskeleton) together with DRAQ5 (nuclei) it became obvious, basically as expected, that the attached cells are concentrated at the interface between the mat and the underlying wound area (Figure 8F-H). The external surface of the mat facing the environment was free of cells (Figure 8B-D).

For the analysis of the cell arrangement after an extended treatment with the mat, for 7 days (after mat change), wound biopsies were taken, which included both a small area of wound regenerating tissue and the collagen mat (Figure 8I). Slices were cut from the immediate wound directed interface mat-wound region and from the subsurface region, approximately 250 µm further to the center of the mat. They were stained with rhodamine/phalloidin together with DRAQ5 and also the (sc-8018) pan-cytokeratin antibody. The images show that not only the wound-directed surface (Figure 8M and N) but also the 250 µm inward area of the mat (Figure 8K and L) lights up with red fluorescence light, reflecting a dense arrangement of the filaments within fibroblasts, more specific myofibroblasts, which characteristically occurs in the granulation tissue; they are involved in wound contraction 39. The myofibroblasts comprise ruffled membranes and possess bundles of microfilaments which fuse together to form an adhesion complex, the fibronexus, at the surface of granulation tissue. Importantly, no signals from potentially present keratins were detectable (should have lighted up in green); Figure 8K to N. As a control, A549 adenocarcinomic human alveolar basal epithelial cells were stained in parallel, which are positive for keratin (green fluorescence) but are lacking a filamentous actin net (red fluorescence) 40; Figure 8J.

### Presence of immune cells in the granulation tissue

During wound healing primarily activated myofibroblasts, contracting the granulation tissue, accumulate as well as organize the repairing cells within the regenerating tissue 41. Subsequently, re-epithelialization by keratinocytes proceeds during which the wound size is reduced and a permanent scar is developed 42. Therefore, the wound region was screened for these two cell types (Figure 9); mostly myofibroblasts were present.

In the first set of experiments sections through biopsies (three weeks after starting the treatment with “Ma/Col-Ca-polyP-NP”) were performed and stained first with hematoxylin/eosin (Figure 9A and B). With this color combination the dense accumulation of cells is visualized in the granulation tissue. The cells are particularly dense at the interface between the regenerating tissue and the collagen mat. The subsequent immune-staining with the cytokeratin pan antibody MNF116 was negative (Figure 9C and D). No distinct signals could be detected. In contrast, the endothelial cell recognizing CD31 antibody gave a distinct positive cell staining (Figure 9E and F), which especially marks the cells that form the microvessels (Figure 9G).

In a comparative analysis a further keratin antibody (anti-AE1/AE3) and an antibody reacting with blood immune cells, like macrophages, platelets, megakaryocytes, dermal dendritic cells, and also fibroblast-like mesenchymal cells (factor XIII polyclonal antibody 43) were applied. Again, the keratin anti-AE1/AE3 staining of the granulation tissue was negative (Figure 9H to J), especially if compared with the adjacent epidermis/dermis layer. In contrast, the staining pattern with anti-factor XIII antibodies gave a speckled distribution of the positive cells. The abundance was relatively low, suggesting that the factor XIII positive cells represent immune cells (Figure 9K to M). It is also interesting that these cells accumulate within the mat (Figure 9M).

## Discussion

Chronic wounds, such as venous ulcers and diabetic wounds, pose a significant burden to patients as well as to healthcare professionals and healthcare systems (reviewed in: 44). While acute wound healing proceeds through the normal phases of wound healing, hemostasis, inflammation, proliferation and maturation, chronic wounds do not pass through this programmed repair pathway due to an unbalanced milieu of growth factors and cytokines, e.g. transforming growth factor-β 45. In addition to impaired inflammatory cytokine levels, vessel insufficiencies (venous or arterial insufficiencies) are involved in the pathogenesis of non-healing chronic wounds 46. As a consequence, angiogenesis is impaired in all chronic wounds, which causes severe tissue damage due to chronic hypoxia and reduced micronutrient delivery. At the biochemical level a severe impairment of enzyme activities of energy metabolism of granulation tissues in regenerating wounds was found both in immunocompromised and in aged rats 47. Ultimately, the ATP level in chronic wounds drops and needs supplementation through exogenous ATP-vesicles 18,48. Emerging evidence has been provided that metabolic energy is supplied to wounds also through polyP, especially via the blood platelets 21. Metabolic energy is needed in a series of steps during wound healing, like protein biosynthesis, cell migration and proliferation, antibacterial activity of leukocytes, or signal transduction and synthesis of growth factors.

It has been found that cells, which expose ALP and ADK, catalyze ATP synthesis in the extracellular space 21, such as SaOS-2 and HUVEC 25,27. Also the cell surfaces of keratinocytes are associated with ALP 49 and ADK 50, the two enzymes which mediate ATP synthesis 21. The polyP polymer was fabricated together with Ca^2+^ in the form of Ca-polyP-NP with a size of around 100 nm 24. The bio-inspired manufacturing process mimics nature; there, especially in the blood platelets, polyP is present as similarly sized NP 51-53. The polyP-NP and also the soluble polyP are released from platelets and channeled into the circulating blood. Accordingly, the Ca-polyP-NP can be homologized with the “extracellular vesicles [ectosomes]” which are likewise released from the cells and contain cell-derived materials in a size range of 30-150 nm 54. These particles are likewise taken up by the recipient cells to elicit their biological effects 55. The polyP that is transferred by the NP used here has a size-range of around 40 P_i_ units; this short-chain polymer displays, if at all and in contrast to long-chain polyP, only a small effect on blood clotting 51,56.

In the present study polyP, as particulate Ca-polyP, was integrated into a wound dressing, fabricated with collagen as a matrix, as described before, or, together with soluble Na-polyP into the wetting solution 19. In preceding studies the collagen matrix has been compressed in order to mimic the nascent wound bed 57 and to increase the stability of the fabricated mats or sheets without losing their flexibility 58. This matrix was proven to provide a suitable substrate for the cells to adhere/attach 19,57, but failed to allow the cells sufficiently to invade into the collagen. Therefore, we developed in the present study a more fluffy collagen scaffold, by reducing the pressure during compression from 50 g to 20 g per sheet of a diameter of 50 mm, and shortened the duration of the process from 60 min to 20 min. The amorphous polyP nanoparticles were prepared with Ca^2+^ as the counterion since in this salt of polyP accelerates the migration activity 27. The concentration of the Ca-polyP-NP in the mats was adjusted to ~8 mg/mL. This formulation was found to increase the growth rate of HUVEC, as measured by the PrestoBlue assay, and their migration activity, as proven in the wound scratch assay.

The fabricated polyP-supplemented collagen mats were not only biocompatible and bioactive for HUVEC, but also for keratinocytes. These two properties are crucial for an effective wound dressing since they support the re-epithelialization process of the keratinocytes 59. In addition, such a mat allows these cells to invade into the collagen matrix. By this, the keratinocytes can grow outward from the lower layers of the dressing and would support cell shedding and replacement of the dead keratinized cells *in vivo* 60. As shown by ESEM microscopic data keratinocytes immigrate into “Ma/Col-Ca-polyP-NP” but not into polyP-free “Ma/Col” and adhere to the collagen fibers. Indicative is also that the polyP-containing mats induce the formation of extended microvilli, while the cells on the “Ma/Col” are decorated only with short such membrane protrusions. Long microvilli provide the keratinocytes with the property to control the fluid diffusion of low-molecular-weight compounds into and out of the cells 36.

Proof-of-concept clinical studies with polyP-enriched mats were performed to treat chronic wounds in patients. Two representative cases with “Ma/Col-Ca-polyP-NP” are shown here. In preceding animal experiments with diabetic mice it was learned that simple application of particulate “Ca-polyP-NP” to an open wound already causes a very significant acceleration of the healing process 20. This effect is attributed to the transition of the NP into the coacervate phase and the ready release of polyP with its morphogenetic potential 61. This process is seen at the interphase between the mats and the regenerating tissue in the wound area. The opposite plane of the mat (directed to the air environment) shows no signs of coacervation, as proteins that mediate the transformation into this phase are missing. The cells that attach to the mats are myofibroblasts, based on their morphology, the development of a fibrillar system in the cytoplasm, and the frayed cell surface. This cell type is dominant during the first phases of wound regeneration; they are highly mobile and provide a platform and support for the migration of the differentiating cells in the wound bed 62.

Biopsies were taken from the regenerating wound, three weeks after starting the treatment with “Ma/Col-Ca-polyP-NP”. During this period vigorous cell proliferation, myofibroblast migration and granulation tissue formation occur, signs which are characteristic for physiological wound healing 6. In the biopsy studied, myofibroblasts appear to be the dominant cell type in the granulation tissue in the regenerating wound area. Approaches to identify keratinocytes by immunostaining for keratin, using the pan-cytokeratin antibody (C11) or the anti-cytokeratin AE1/AE3 antibody were not successful. In addition to myofibroblasts a distinct staining of cell clusters with a CD31 (PECAM-1) antibody lighted up, which also stained the cells around the rarely present microvessels. Finally, Factor XIII positive cells were found more abundantly in the granulation tissue area and in smaller nests within the mat. Based on the comparably low frequency of the cells in this area it appears that they reflect blood immune cells and not fibroblasts. Also the time of the occurrence of the immune cells matches with the wound healing period, which is limited to 3 to 7 days, with the inflammation phase 6.

An efficient wound care during the phases of hemostasis, cell proliferation and remodeling needs the provision of a humid environment. Therefore, the mats were regularly (daily or at least every other day) moisturized with a wetting solution supplemented with soluble Na-polyP and particulate “Ca-polyP-NP”. By this, polyP is immediately available through the Na-polyP and phase shifted, in a depot form, to “Ca-polyP-NP”. The effect of this application is a two-fold; first to supply a humid environment to the wound and second a replenishment of the regeneration zone with the active component polyP.

## Conclusion

**Figure 10:**
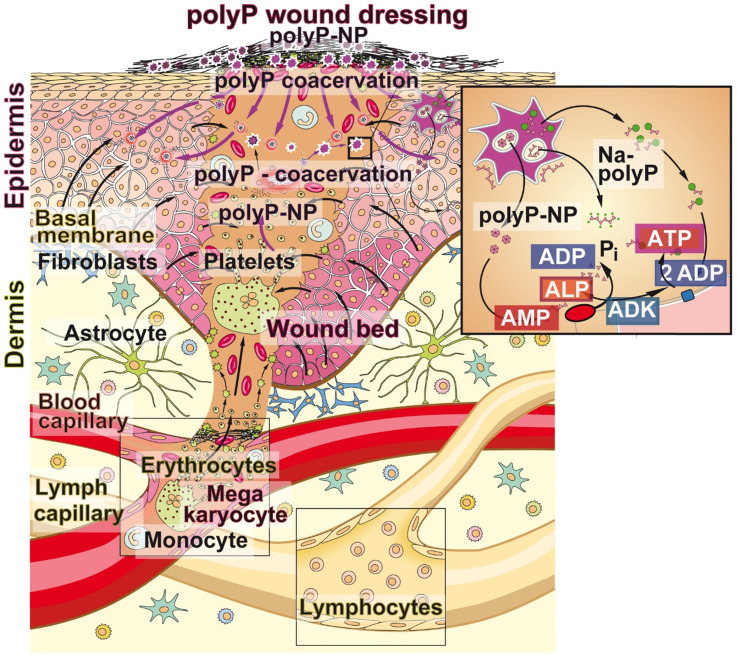
** Bidirectional supply of polyP to the regenerating wound bed (scheme). *First source of polyP***; the physiological delivery *via* the circulating blood system through the blood platelets that originate from megakaryocytes and accumulate in the damaged tissue area. There, and also in the wound exudate, ALP is present, which degrades polyP under release of metabolic energy that is stored in ADP (insert). The ADK which converts ADP to ATP and AMP is associated with blood cells. ***Second source of polyP - from the mat***; the wound dressing is additionally supplemented with polyP, “Ma/Col-Ca-polyP-NP”, in a depot form as nanoparticles to the wound. Finally, polyP is drizzled onto the mat with the wetting solution “WetSol”, containing both soluble Na-polyP and particulate “Ca-polyP-NP” (not shown in the scheme).

PolyP is a natural polymer that is spread via the blood circulation system through the body and accumulates especially at those sites where tissue repair processes occur. The polymer is present at higher concentrations in blood platelets which adhere to the extracellular matrix very early during the hemostasis phase. In addition to the release of polyP from the platelets, the activated platelets and the damaged endothelial cells release ADP 18. Since ALP is abundantly present in serum but also in the wound exudate 63 polyP is prone to rapid enzymatic hydrolysis to orthophosphate with simultaneous synthesis of ADP and then, in concert with the ADK, which is present on the wound/skin cell surfaces, to a conversion of ADP to ATP and AMP 21. Metabolic energy in the form of ATP is required to supply the cells in the regenerating wound for their cell proliferation. In turn, polyP is supplied to the regenerating area in two routes, first via the bloodstream, and second, as demonstrated here for chronic wounds, via the polyP-enriched wound dressing (Figure 10). Based on the conclusive healing data and first clinical assessments, summarized here, it is proposed that addition of polyP, both in the form of soluble Na-polyP and as particulate Ca-polyP nanoparticles which act as a depot form, is a promising therapeutic approach to successfully treat chronic wounds. The presented “bench to beside” approach underscores a successful example for a translational research activity starting with encouraging pre-clinical data which have been channeled to the clinics. The first beneficial clinical applications are summarized here. In turn, larger clinical studies are strongly recommended.

## Materials and Methods

### Materials

Na-polyphosphate (Na-polyP) with an average chain length of 40 phosphate (P_i_) units was purchased from Chemische Fabrik Budenheim (Budenheim; Germany).

### Preparation of Ca-polyP-NP

Amorphous nanoparticles were prepared as described 24,64. Na-polyP (1 g) was dissolved of in 25 mL of distilled water and added dropwise to 3.86 g of CaCl_2_·6H_2_O (#A537.1; Roth, Karlsruhe; Germany) also dissolved in 25 mL of distilled water; the pH was adjusted to 10. After stirring for 12 h the particles formed were collected by filtration, washed with ethanol and dried at 50 °C. They are termed “Ca-polyP-NP”.

### Preparation of collagen-based mats

As a source for collagen, bovine collagen solution (type I) was prepared from bovine tendon collagen type I (Lando Biomaterials, Shenzhen; China). The collagen solution was dissolved in 0.01 N HCl (pH 2.8) 19,65. An aliquot (8 mL) was placed into a plastic Petri dish (diameter of 50 mm) and kept at 4 °C. After standing on ice for 2 h, the collagen sample (10 mg/mL with respect to collagen) was neutralized with a sodium hydroxide solution (1 M) and subsequently incubated overnight at 37 °C (5% CO_2_ atmosphere); “Ma/Col”. The pH shift was included in the fabrication process in order to accelerate an organized fibril formation 66.

In parallel, 8 mg of “Ca-polyP-NP” were added to 1 mL collagen solution and processed; “Ma/Col-Ca-polyP-NP”. Both types of mats, “Ma/Col” and “Ma/Col-Ca-polyP-NP” were washed twice with PBS (phosphate-buffered saline). This solvent was then replaced by ethanol (70% v/v). The respective gel pad was removed and laid onto a filter paper stack (Whatman-3MM-blotting paper). After putting on top a nylon mesh filter (100 μm) a weight of 20 g was put on the stack for 20 min to squeeze the layer to a thickness of 1 to 1.3 mm. Then the mats were stored in 70% (v/v) ethanol.

### Cultivation of cells

The human epidermal keratinocytes were purchased (#102-05A, Sigma-Aldrich). The cells were cultivated in complete epidermal keratinocyte culture medium (#SCMK001, Sigma-Aldrich), as described 67,68, and plated into 24-well plates coated with 200 µL of either polyP-free “Ma/Col” or polyP-containing “Ma/Col-Ca-polyP-NP”. Onto the 1 mm thick layer 4×10^4^ cells in 1 mL culture medium were added. As indicated, the cells were incubated for 24 h and then inspected by environmental scanning electron microscopy (ESEM).

Human umbilical vein endothelial cells (HUVEC), obtained from Lonza (Basel; Switzerland), were incubated in endothelial growth medium with 4% v/v fetal bovine serum (FBS; #F7942, Sigma) and 0.4% bovine brain extract (BBE; #CC-4098, Lonza) 26. Usually the tissue culture test plates were coated with poly-L-lysine solution (#A-005-C, Sigma) as outlined 69.

In one series of experiments A549 cells were used as a control for a panel of the intermediate filament keratins 70. The cells were cultivated as described 71.

### Cell growth and proliferation assay

For the determination of cell growth/proliferation of HUVEC, the cells were seeded onto a hydrogel layer and incubated for 48 h or 72 h in 24-well plates. Then the cells were reacted in the colorimetric assay with PrestoBlue (#A13261; Invitrogen/Thermo Fisher Scientific, Dreieich; Germany) as described 72,73. In brief, the cells were incubated with the PrestoBlue reagent and after lysis of the cells, the change of the color caused by the reduction of resazurin to resorufin was measured at a wavelength of 560 nm and 620 nm. Ten parallel experiments were performed; data are means ± SD (*p < 0.01).

### Wound scratch assay

The cells (HUVEC; 3×10^5^ cells per dish) were seeded into 6 cm Petri dishes, covered with a 1 mm thick layer of either “Ma/Col” or “Ma/Col-Ca-polyP-NP” until a confluent monolayer was reached. Then, this layer was scratched with a 200 µL pipette tip. After washing the cultures were continued to be kept for 8 h or 24 h and then inspected for wound repair using a light microscope 74. The area within the scratch zone, which was covered with HUVEC, was assessed with the WimScratch software (Córdoba; Spain), as described 75.

### Preparation of the wetting solution

A 120 mM phosphate buffer (pH 6.5) was adjusted with NaCl to 80 mM. Then the solution was supplemented with 20% (v/v final) propylene glycol (#398039, Sigma-Aldrich) to enrich the solution with antimicrobial and antifungal quality 76. Finally, 300 µg/g Na-polyP and 30 μg/g (w/w final) Ca-polyP-NP were added. The final wetting solution was sterile-filtered (Sterifix 0.2 µm; B. Braun, Melsungen; Germany); “WetSol”.

In one series, a “Ma/Col-Ca-polyP-NP” sample (diameter of 50 mm) was treated with 1 mL of “WetSol” solution and then kept in a humid chamber for 6 h prior to scanning electron microscopy (SEM) analysis.

### Application for chronic wounds

An off-label use/application (after written and signed informed consent) was performed with five patients. Among them, two patients are depicted here. The 79-years old male patient suffered from an ulcer which covered the ventral tibia of the left lower leg. It persisted for 1.5 years and is considered as a secondary disease which occurred in the course of a follicular lymphoma. The chemotherapy started at 02/2019 with a maintenance therapy with Obinutuzumab and Dexamethasone. In 01/2020 the therapy included Clemastine. After resection of a squamous cell carcinoma which appeared in 2/2020 the wound was covered 3-times with a split-thickness skin graft without healing. With the consent of the patient the therapy treatment with the “Ma/Col-Ca-polyP-NP” started in April/March 2021 following the rules of Good Clinical Practice and the declaration of Helsinki.

The second patient, an 82 old woman was diagnosed with an ulcer. This therapy resistant traumatic open wound (ulcer) formed on the left lateral malleolus in 6/2020. Peripheral arteriosclerosis as well as chronic venous insufficiency could be excluded. Despite various standard therapies, the ulcer did not heal and the patient was presented in March 2021 with a redness around the wound, severe pain and intense signs of inflammation. After signing the informed consent, the therapy with polyP mats was initiated.

Where indicated, the wound area was measured after taking images. They were circled and then sized with the ImageJ program 77 after lengths calibration. The areas are given in cm^2^.

### Removal of a tissue excision sample and clinical course

Necrotic tissue and epithelial debris were removed by surgery and ultrasound (UAW instrument; Söring, Hamburg; Germany). After three cycles of wound cleaning, a collagen mat enriched with polyP, “Ma/Col-Ca-polyP-NP”, was applied on the wound. Replacement of the wound dressing was performed every second or third day mainly following the patient's instructions. Then, one mL of the polyP supplemented wetting solution, “WetSol”, was dropped onto the mats and fatty gauze was overlayed to prevent dehydration. After three weeks of treatment and a striking reduction of the wound area, a punch biopsy was taken to follow histologically the clinical wound healing process.

### Histology - Cell distribution in the wound dressing

The distribution and the typing of cells within the mat used during the therapy were studied in the collagen-based wound dressing “Ma/Col-Ca-polyP-NP” three weeks after starting the treatment. Re-epithelization was assessed on the basis of a reduction of the wound edges, resulting in a circular lifting of the mat from the border. These physiological overhangs were used for the analysis of the distribution of the cells within the mats. Both the side facing the environment (outside) and the interface surface to the wound (inside) were studied. The aim was also to visualize the polyP-coacervate formation at the inside and its lack on the outside by ESEM.

In addition, the abundance of cells was visualized by rhodamine/phalloidin to illustrate F-actin of the cellular cytoskeleton in concert with the rhodamine fluorescent marker. Approximately 250 µm thick slices were removed from the outside and from the inside/wound side of the mat. In addition, a sub-interface layer was sectioned-off, approximately 100 µm apart from the wound edges. The specimens were fixed in 4% neutral and buffered formalin and subsequently permeabilized with acetone. After blocking with 5% bovine serum albumin (#B4287; Sigma-Aldrich) the samples were stained with rhodamine/phalloidin (#R415; Invitrogen/Thermo Fisher Scientific) to document the organization of the cytoskeleton (in red). In addition, the samples were reacted with DRAQ5 (#62251; Thermo Fisher Scientific) to visualize the nuclei (blue). Finally, they were stained with the (sc-8018) pan-cytokeratin antibody (C11) (Santa Cruz; CA) and the corresponding isotype control antibody (sc-3890; Santa Cruz; CA), respectively. As a keratin positive control A549 cells were used. The specimens were inspected with an EVOS fluorescence microscope (Invitrogen/Thermo Fisher Scientific, Dreieich; Germany).

For the visualization of the F-actin cytoskeleton the emission wavelength was 585 nm and that for excitation was 535 nm; the nuclei were inspected at an emission 665 nm/excitation 647 nm. The keratins were depicted at emission 525 nm/excitation 490 nm.

### Histology - Cells in the biopsy

For histological inspection the surgically excised tissue samples were fixed in paraformaldehyde (#4760; Sigma) and embedded into paraffin wax (#03987; Sigma). The paraffin tissue blocks were sectioned into *~*5 µm thick sections and mounted onto glass slides (microscope slides; #XX1007615; Sigma-Millipore) 75. Then the specimens were deparaffinized followed by rehydration through the ethanol series.

The sections were stained with hematoxylin/eosin, using Mayer's hematoxylin (#MHS1, Sigma-Aldrich) and eosin Y solution (#HT110280; Sigma-Aldrich) as described 78. The potential reactivity towards cytokeratin-5, -6, -8, -17 and -19 was screened with the cytokeratin pan antibody MNF116, a mouse/IgG1 (#MA1-26237; Invitrogen/Thermo Fisher Scientific), reacted with an ALP-labelled anti-mouse IgG secondary antibody (GtxMu-004-EALP; Dianova GmbH, Hamburg; Germany). Immuno-staining for keratin AE1/AE3 was performed with anti-cytokeratin AE1/AE3 antibody (#MAB3412 - clone AE1/AE; Sigma-Aldrich) in combination with an anti-mouse secondary antibody, labelled with ALP (GtxMu-004-EALP; Dianova). The immunocomplexes were visualized with the ALP-substrate mixture nitro blue tetrazolium chloride (NBT; #N5514; Sigma) and 5-bromo-4-chloro-3-indolyl phosphate (BCIP; #B6149; Sigma). CD31 (PECAM-1) is present in larger quantities on the surfaces of endothelial cells, thrombocytes, monocytes and in granulocytes and is screened in the samples with the CD31 (PECAM-1) monoclonal antibody (#12-0311-82; Invitrogen/Thermo Fisher Scientific). Again, the complexes were highlighted with an ALP-labelled anti-mouse IgG (GtxMu-004-EALP; Dianova). The Factor XIII polyclonal antibody (#PA5-16423; Invitrogen/Thermo Fisher Scientific) was applied together with a horseradish peroxidase labelled secondary antibody (SBA-4090-05; Dianova). The immunocomplexes were stained with the substrate-chromogen PolyDetector AEC HRP kit (Bio SB; Santa Barbara; CA).

The specimens were inspected with a phase contrast/DIC microscope (Olympus, Hamburg; Germany).

### Microscopic analysis

The ESEM observations were performed with a Philips microscope (Eindhoven; Netherlands) and the SEM studies with a Zeiss Gemini 1530 (Zeiss Oberkochem; Germany). For the ESEM analyses the cells were fixed in 2% [v/v] aqueous glutaraldehyde and then treated with osmium oxide 79. Finally, the specimens were processed through acetone dehydration steps and subjected to critical point drying at 43 °C 75. Light microscopical images were taken with a VHX-600 Digital Microscope (Keyence, Neu-Isenburg; Germany).

### Statistical analysis

For the statistical analyses the Student's *t* test was applied. The average values came from ten independent experiments, if not stated otherwise. Values of *p* < 0.01 were considered statistically significant (*). The calculations were performed with the GraphPad Prism 7.0 software (GraphPad Software, La Jolla, CA, USA).

## Abbreviations

ADK: adenylate kinase
ALP: alkaline phosphatase
ATP: adenosine triphosphate
Ca-polyP-NP: calcium polyphosphate nanoparticles
EGF: epidermal growth factor
ESEM: environmental scanning electron microscopy
HUVEC: human umbilical vein endothelial cells
Ma/Col: collagen fiber mat
Ma/Col-Ca-polyP-NP: collagen fiber mat with Ca-polyP-NP
NP: nanoparticles
polyP: polyphosphate
SaOS-2: human bone osteosarcoma cell line
WetSol: wetting solution
